# Characterisation of a Novel *Pseudomonas* Phage and Its Effect on the Survival of *Galleria mellonella* Larvae

**DOI:** 10.3390/pathogens14121248

**Published:** 2025-12-06

**Authors:** Sukran Ozturk, Hilal Basak Erol, Banu Kaskatepe, Wan-Ting Huang

**Affiliations:** 1 Department of Pharmaceutical Microbiology, Faculty of Pharmacy, Zonguldak Bulent Ecevit University, 67100 Zonguldak, Turkey; 2 Department of Pharmaceutical Microbiology, Faculty of Pharmacy, Ankara University, 06100 Ankara, Turkey; bkaskatepe@ankara.edu.tr; 3 Precise Health SA, C/O The Ark Foundation, Rue de L’Industrie 23, 1905 Sion, Switzerland; wan-ting@precisehealth.io

**Keywords:** bacteriophage, phage–antibiotic synergy, synergy, checkerboard method, *Galleria mellonella*

## Abstract

Multi-drug-resistant *Pseudomonas aeruginosa* (*P. aeruginosa*) commonly causes infections that are difficult to treat, necessitating the development of new therapeutics. The search for more effective ways to combat the emergence of bacterial resistance has also led to research into phage-antibiotic synergy (PAS) as a potential therapeutic strategy. The aim of this study was to isolate and characterize virulent phages from water sources that are active against clinical carbapenem-resistant *P. aeruginosa* isolates, and to evaluate their in vivo efficacy using a *Galleria mellonella* larvae infection model. The biological and genomic characteristics of the isolated phages were determined using host range analysis, one-step growth curve analysis, transmission electron microscopy analysis and whole-genome sequencing. Two phages (vB_PaMB13 and vB_PaMB17) that demonstrated in vitro synergistic and bactericidal interactions with antipseudomonal antibiotics (tobramycin and ceftazidime) were selected for further investigation using the checkerboard method. The study revealed synergy between all phages and either antibiotic, tobramycin or ceftazidime, against *P. aeruginosa*. Similarly, the percentage survival rates increased in the in vivo model when both phages and antibiotics were used in combination. Overall, our study provides further support for the idea that phage-antibiotic synergy could be an effective strategy for improving treatment outcomes.

## 1. Introduction

*Pseudomonas aeruginosa* (*P. aeruginosa*) is listed as a high-priority bacterial pathogen on the 2024 WHO Bacterial Priority Pathogens list and poses a significant global health threat [1,2]. The fight against *P. aeruginosa* also requires the implementation of effective eradication procedures. However, the tendency of *P. aeruginosa* to develop resistance to a wide range of antiseptics, disinfectants, and antibiotics makes this difficult and reduces success rates. This failure has led to increased mortality rates in severe infections, higher costs and longer hospital stays, particularly in hospital-acquired infections [3,4].

The most commonly prescribed antibiotics for *P. aeruginosa* infections are beta-lactams, fluoroquinolones and aminoglycosides [5,6]. The use of bactericidal aminoglycoside antibiotics was initially successful, but the first cases of resistance were reported in the 1960s. *P. aeruginosa* can develop resistance to aminoglycosides by several mechanisms: lipopolysaccharide (LPS) modifications (mediated by the PhoPQ two-component system), ribosomal changes (caused by 16S rRNA ribosomal methyltransferases), production of antibiotic-inactivating enzymes, and efflux pump systems [7,8]. They are also known to have synergistic effects with β-lactam antibiotics. They are widely used to treat lung infections, especially in patients with cystic fibrosis (CF), preventing the development of resistance and increasing efficacy [9,10,11].

Carbapenems (imipenem, meropenem, and doripenem) have been the antibiotics of choice for multidrug-resistant *P. aeruginosa* infections since the 1980s. Carbapenem resistance mechanisms in *P. aeruginosa* are multifactorial and result from interactions among factors including mutations in the outer membrane protein OprD, overexpression of the efflux pump MexAB-OprM, production of acquired Amber class B metallo-β-lactamases (MBLs), and overproduction of chromosomal β-lactamase (AmpC) [12].

Bacteriophages are viruses that infect and replicate within bacterial cells, commonly referred to as phages [13]. Scientific interest in obligately lytic bacteriophages has surged due to their potential applications in various fields. Phage therapy, which involves using phages to treat bacterial infections, has become a promising alternative to antibiotics. Several clinical trials are underway to evaluate its effectiveness in treating conditions such as cystic fibrosis, sepsis, and antibiotic-resistant wounds [14]. FDA-approved phages are currently used as Emergency Investigational New Drugs (eINDs) to treat MDR bacteria, and in Europe, local authorities in some countries have granted permission to use phages in the “compassionate use” category [15,16].

*G. mellonella* larvae are a frequently used alternative in vivo model for infection studies. The immune system of *G. mellonella* shares similarities with the innate immune system of mammals [17]. They are preferred as an intermediate step before in vivo experiments because they can survive at 37 °C, are inexpensive and readily available, are easy to maintain, and do not require ethical approval [18]. Furthermore, infection assays provide results within 24–48 h and have high sensitivity for human pathogens; these are additional advantages of this method [19]. The *G. mellonella* model is valuable for preliminary screening and virulence factor assessments for human infections, but due to the lack of adaptive immunity, lack of mammalian organ and tissue structures, and different pharmacokinetic/pharmacodynamic profiles, the results cannot fully predict human diseases [20].

Numerous studies have examined *Pseudomonas* phages in the literature. For example, Nour El-Din et al. (2025) isolated broad-spectrum phages against cystic fibrosis isolates and found that some prolonged the lifespan in the *Galleria mellonella* model [21]. Similarly, a study of biofilm activity found that the *Pseudomonas* phage named ‘Motto’ disrupted biofilms produced by strong biofilm-forming strains and resulted in 90% survival of *Caenorhabditis elegans* in the model [22]. In a mouse bacteraemia model, a single dose of two different phages completely eliminated *P. aeruginosa* bacteraemia and result in 100% survival of the mice [23]. While some studies have reported the effectiveness of monophages or phage cocktails used in combination with antibiotics, there is still insufficient research on the use of phage therapy in human patients [24].

In empirical antimicrobial treatment, combination therapy may be used. Reports indicate that phage-antibiotic combinations are more effective in treating *P. aeruginosa*, which has been the focus of in vitro studies due to its clinical importance. PAS trials have investigated the in vitro bacterial suppressive activity of cefotaxime, ciprofloxacin, gentamicin, meropenem, tetracycline, and ceftazidime [25,26,27]. Combined therapy is expected to use lower doses of phage and antibiotics resulting in increased combined efficacy. Additionally, it has been demonstrated that combining antibiotics and phages increases the number of phages produced during infection [28].

In our study, *P. aeruginosa*-specific lytic phages were isolated and characterized, and the two most effective phages were selected. The synergistic and bactericidal interactions of these two phages, which were selected as candidates, with antipseudomonal antibiotic classes were demonstrated in vitro using the checkerboard method and in vivo in the *Galleria mellonella* larvae model, respectively.

## 2. Materials and Methods

### 2.1. Bacterial Strains

In total, 30 non-duplicate clinical multi-drug-resistant *P. aeruginosa* isolates were obtained from hospitals (Ankara, Turkey). All bacterial isolates used for the determination of phage activity. These isolates were cultured in Trypticase Soy Agar (TSA, Merck, Darmstadt, Germany) at 37 °C for one night. The synergistic activity of phage and antibiotic was tested by selecting three strains. Among the resistant strains, three randomly selected strains with MIC values close to the high, moderate, or borderline were selected to represent each profile (P6, P12, P24). For the in vivo model, all three isolates were used. Two classes of antibiotics were tested, including, beta-lactams (ceftazidime) and aminoglycosides (tobramycin) which were purchased commercially from Sigma Chemical Company (St. Louis, MO, USA).

### 2.2. Isolation, Purification, and Concentration of Bacteriophages

The bacteriophages specific to *P. aeruginosa* were isolated from hospital and wastewater sources in Ankara, Turkey. The double-layer agar method was employed to detect the presence of phages in the samples following phage enrichment as described in our previous study [29]. After incubation, the Petri dishes were analyzed for the presence of phage plaques. Single plaque isolation was performed for purification in Petri dishes with bacteriophage plates [30]. A concentrated phage suspension was prepared for phage plates that were considered to be pure. The phages were propagated with a host bacterium at 37 °C for one night in Luria–Bertani broth (LB, Sigma, St. Louis, MO, USA). After propagation, the phage-bacteria culture was centrifuged at 9000× *g* for 10 min and then filtered through 0.22 µm membrane filter. The filtered phages were titered using the double-layer agar method [31]. In brief, the concentrated phages were serially diluted in phosphate-buffered saline (PBS; pH 7.2). Diluted phage solutions were mixed with host cells (log phase bacteria) in soft agar (containing 0.8% agar in LB broth) and then poured onto the LB agar plate. After one-night incubation at 37 °C, the plaques were enumerated as plaque-forming units (PFU/mL). A high-titer phage lysate was precipitated using polyethylene glycol 8000 (10% *w*/*v*) at 4 °C overnight and centrifuged at 12,000× *g* for 20 min at 4 °C. Then, the pellet was resuspended in SM buffer.

### 2.3. Determination of the Host Range

From the fresh cultures of the hospital isolates (10^8^ CFU/mL), the bacterial suspension was streaked on an agar plate and 10 µL of phage suspension (10^8^ PFU/mL) was dropped in these areas. Following overnight incubation, the areas where no growth was observed in the sowing areas were evaluated [32].

### 2.4. Transmission Electron Microscopy

Imaging was performed using a transmission electron microscope to determine the morphological characteristics of the selected bacteriophages. Bacteriophages were first negatively stained with uranyl acetate (2%) and were then imaged using a G2 S Twin 200 kV RTEM (FEI Company, Bellaterra, Spain) transmission electron microscope at 120 kV on a formvar-coated carbon grid [33].

### 2.5. One-Step Growth Curve

The bacteriophage and the host bacterial culture were mixed to reach an MOI of 0.1. The mixture was incubated for 15 min at 37 °C to allow for complete adsorption and then centrifuged for 1 min at 15,000× *g* to remove unabsorbed bacteriophages by discarding the supernatant. The pellets were resuspended in LB broth and incubated at 37 °C. Taking 100 µL of the sample every 10 min, the process continued until the 90th minute. Free bacteriophages were titrated after centrifugation at 15,000× *g* for 1 min to remove bacterial cells from the samples [34].

### 2.6. Whole Genome Sequencing

Phage DNA was extracted using MN NucleoSpin Virus DNA/RNA Purification Kit (Cat no: 740983.50). The high-sensitivity dsDNA kit (Invitrogen, Waltham, MA, USA) was used to quantify phage DNA using a Qubit 3.0 fluorometer. An Illumina DNA Prep kit was used to prepare the phage library. The library was quantified on a Qubit 3.0 fluorometer with the use of the high-sensitivity dsDNA kit. The phage library was sequenced on the Illumina NextSeq 500 platform, producing 2 × 150 bp paired-end reads for whole genome sequencing.

For bioinformatic analysis, quality control of paired-end sequencing reads was performed using fastp (v0.24.0) with default parameters, including the -detect_adapter_for_pe option to remove adaptors and low-quality reads [35]. Trimmed reads were assembled with the Shovill (v1.1.0) program, applying filtering thresholds of 500 bp for minimum contig length (–minlen) and 10× for minimum contig coverage (–mincov). To assess assembly quality, detect host contamination, and identify proviruses, CheckV (v1.0.3) end_to_end program was run with default settings [36]. The functional and structural genome annotation was carried out using Pharokka (v1.7.5) with default settings [37]. The annotation included predicted coding sequences (CDSs), predicted genes and proteins, and the closest known phage to our genome, etc. Genome map showing the positions of CDSs and the predicted proteins was generated with the website-based tool, Proksee [38].

For taxonomic assignment, a subset of the reference genomes from the International Committee on Taxonomy of Viruses (ICTV) release (MSL39.v4) was selected for phylogenetic analysis using the VIRIDIC web (https://rhea.icbm.uni-oldenburg.de/viridic/ (accessed on 22 January 2025). The subset includes (i) exemplar genomes from the same genus as the closest phage identified by Pharokka, (ii) exemplar genomes from other genera within the same family, and (iii) exemplar genomes from a different family as outgroups. In addition, the pairwise comparison was calculated using FastANI (v1.34) with default settings [39].

### 2.7. Checkerboard Assay

After screening three multi-drug resistance *P. aeruginosa* strains against two well-characterized *P. aeruginosa* phages were selected for synergy testing based on high phage sensitivity and substantial antibiotic resistance patterns. The PAS was examined using the checkerboard method in a sterile the 96-well plate [40]. Two-fold serial dilutions of ceftazidime or tobramycin (0.25–128 μg/mL) at horizontal rows and each phage separately (10^12^–10^3^ PFU/mL) at vertical rows were prepared. The concentrations of bacterial suspensions were prepared according to 0.5 McFarland turbidity and added to each well so that the final bacterial concentration in the wells was 5 × 10^5^ CFU/mL. Microplates were incubated at 35 °C for 18–20 h. The synergy between antibiotics and phages was quantified by determining the fractional inhibitory concentration index (FICi) using the following formula: FIC of phage = MIC phage when tested in combination with antibiotic/MIC of phage alone, FIC of antibiotic = MIC antibiotic when tested in combination with phage/MIC of antibiotic alone, FICi = FIC_phage_ + FIC_antibiotic_.

The FICi index obtained was interpreted as follows: <0.5 synergy; 0.5–0.75 partial synergy; 0.76–1 additive effect; 1–4 indifference; and >4 antagonisms.

### 2.8. In Vivo Model of Phage Antibiotic Combination

*G. mellonella* larvae were grown from the Zonguldak Bulent Ecevit University Faculty of Pharmacy Pharmaceutical Microbiology Laboratory, Turkey. vB_PaMB13 and vB_PaMB17 phages were used to determine the therapeutic effect on the *G. mellonella* larvae model infected with the P12 strain. All the *G. mellonella* larvae weighing 250–350 mg were selected for the experimental study and maintained on a semi-synthetic diet (150 mL liquid honey, 300 mL glycerin, 500 mg dried bran, 200 mg honeycomb, 150 mL water) at 28 °C in the incubator [41]. P12 bacterial suspension, which was determined in our previous studies, was prepared according to McFarland 0.5 (1.5 × 10^6^ CFU/mL). Ten larvae were used for each group in the study, and the worms were starved for 24 h before starting the experiment. First of all, for evaluating of the therapy dosage of selected phage, larvae were injected with ten μL of phage lysate (10^8^ PFU/mL). Each group was carried out for 96 h at 37 °C, and results were evaluated at 24, 48, 72, and 96 h. The results were expressed as the percentage survival rates and by assessment of survival, melanisation, and macroscopic appearance. The experiments were repeated three times.

Larvae study was designed with lead groups to determine lethal dosage

Group vB_PaMB13 (10^8^ PFU/mL): Healthy control larvae + vB_PaMB13 injection

Group vB_PaMB17 (10^8^ PFU/mL): Healthy control larvae + vB_PaMB17 injection

Following the dose determination study for vB_PaMB13 and vB_PaMB17 phages, working groups were formed. The study was designed in 2 groups (therapy and control group). Treatments (phage and/or antibiotic) were administered 120 min after infection. In combination groups, phage was injected first followed by antibiotic within 1 min. Dose per larva was calculated as follows—Phage: 10 µL at 1 × 10^8^ PFU/mL = 1 × 10^6^ PFU/larva; ceftazidime: 10 µL at 0.3 mg/mL = 3 µg/larva (≈10 mg/kg for a 300 mg larva).

Group A:

Group 1: Healthy Control (phosphate-buffered saline [PBS]

Group 2: P12 infection Control (1.5 × 10^6^ CFU/mL)

Group 3: P12 infected + vB_PaMB13 therapy

Group 4: P12 infected + ceftazidime (0.3 µg/mL) therapy

Group 5: P12 infected + vB_PaMB13 therapy + ceftazidime therapy

Group B:

Group 1: Healthy Control (phosphate-buffered saline [PBS]

Group 2: P12 infection Control (1.5 × 10^6^ CFU/mL)

Group 3: P12 infected + vB_PaMB17 therapy

Group 4: P12 infected + ceftazidime therapy

Group 5: P12 infected + vB_PaMB17 therapy + ceftazidime therapy

### 2.9. Evaluation of Bacterial Growth in Galleria mellonella Larvae

The CFU determination method was used to quantify the number of bacterial colonies in the tissues and hemolymph of larvae infected with isolate P12 and subsequently treated with vB_PaMB13. Five larvae were randomly selected from the five groups examined, and each group was kept in separate Petri dishes. Five randomly selected larvae were treated with 70% alcohol and dissected with a scalpel.

Larvae samples were homogenized in phosphate-buffered saline (PBS) (pH = 7.1) and then treated with 1% Triton X-100 for 15 min to promote bacterial release. Serial dilutions (1/10–1/100,000) were made using PBS and cultures from each dilution were spread onto Mueller Hinton Agar medium. The agar plates were then incubated at 37 °C for 18–24 h, after which colony counting was performed [42,43,44,45].

### 2.10. Statistical Analysis

The experiments were repeated three times. The mean of three experimental results was calculated for each test. The statistical analyses were carried out using SPSS 19.0 software. The Kruskal–Wallis test was used to compare groups for survival function. Primary endpoint was defined as 96 h survival. All larvae (n = 10/group) were included in the analysis. Kruskal–Wallis with Dunn’s post hoc test (corrected for multiple comparisons) was applied. Kaplan–Meier analysis with log-rank test was used for group comparisons. A *p*-value of less than 0.05 was considered statistically significant.

## 3. Results

### 3.1. Isolation and Characterization of Phages

**Figure 1:**
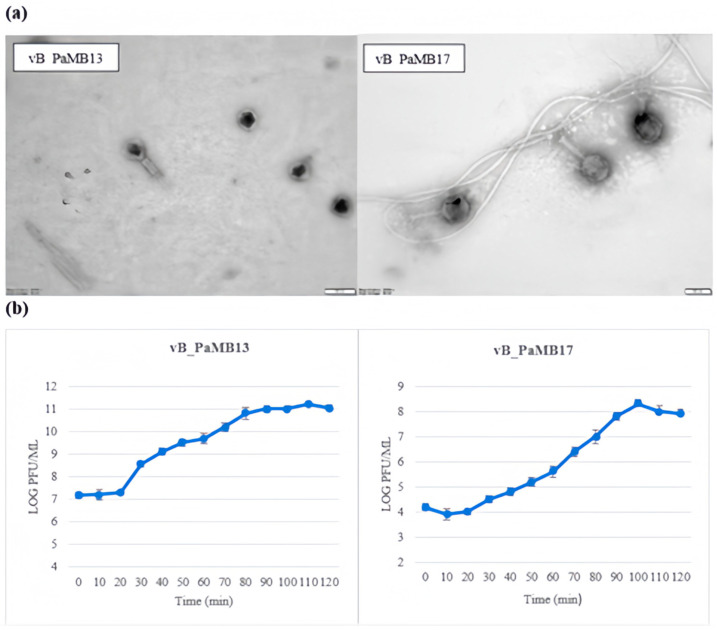
Transmission electron microscopy images (**a**) and the one-step growth curves (**b**) of isolated phages.

A total of 18 *Pseudomonas* phages were isolated from the wastewater of a hospital. The phages were separated based on their different plaque morphologies and varying isolation times. The phages were numbered vB_PaMB1-vB_PaMB18 when obtained as uniform plaques. The lytic activity of the phages against 30 multi-drug-resistant *P. aeruginosa* isolates tested ranged from 16.6 to 93.3% (Table S1). The host range of vB_PaMB13 phage was 93.3 (28/30 strains) and that of vB_PaMB17 was 83.3 (25/30 strains). Among the phages whose host ranges were determined, vB_PaMB13 and vB_PaMB17 were selected for further studies. The selected phages have been characterized. Images taken at 60,000× magnification and at high voltage (80 kV) using a TEM microscope are shown in Figure 1a. vB_PaMB13 was found to have a myovirus morphology with a head of 88 × 72 nm in diameter and a tail of 72 nm in length, and vB_PaMB17 was found to have a myovirus morphology with a head of 125 × 110 nm in diameter and a tail of 130 nm in length. Based on the one-step growth curve results, the latent period for both vB_PaMB13 and vB_PaMB17 phages was estimated as 20 min, while the rise periods were 90 min and 80 min, the burst sizes were 250 and 620 PFU/cell, respectively (as seen in Figure 1b).

Whole genome analysis of vB_PaMB13 phage was studied due to its higher host range. The complete genome of *Pseudomonas* phage vB_PaMB13 was determined to be linear, 66,375 bp in length, with 111 bp direct terminal repeats (DTRs) and a GC content of 56%. Taxonomic analysis assigned vB_PaMB13 to the genus *Pbunavirus*. The closest match reported by Pharokka was *Pseudomonas* phage vB_PaeM_V524, with an average nucleotide identity (ANI) of 98.07%. To validate this classification, we performed VIRIDIC analysis against a curated subset of ICTV reference genomes, including members of *Pbunavirus*, related genera within the same family, and genomes from a different family as outgroups. The VIRIDIC results confirmed that vB_PaMB13 clusters within *Pbunavirus*, showing the highest similarity to three *Pbunavirus* reference genomes (seen in Table S2 and Figure S1. Pairwise comparisons with these genomes using fastANI revealed ~97% nucleotide identity, slightly lower than the similarity observed with vB_PaeM_V524.

**Figure 2:**
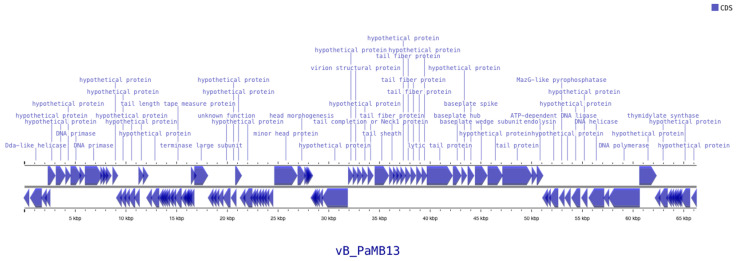
Map of the genome organization of bacteriophage vB_PaMB13 created by using Proksee. The CDSs with predicted annotations are indicated with blue arrows.

To avoid duplication of terminal genes, annotation was performed on the sequence with one copy of the DTR removed. Genome annotation identified a total of 107 CDSs, of which 31 were assigned putative functions and 76 were of unknown function. Functionally annotated genes are displayed in the genome map (Figure 2), which highlights the distribution of CDSs across the genome. Among the functionally annotated genes, one connector gene, eight genes related to DNA, RNA, and nucleotide metabolism, six genes involved in head structure and DNA packaging, one lysis gene, and fourteen tail-associated genes were identified. No genes related to integration/excision, transcription regulation, auxiliary metabolic functions, tRNAs, tmRNAs, or CRISPR elements were detected. Importantly, no antimicrobial resistance (CARD) or virulence factor (VFDB) genes were present in the genome, further supporting the safety of this phage for therapeutic applications.

### 3.2. The Results of the Checkerboard Assay of the Phage-Antibiotics Combination

The stock concentrations of the antibiotics (ceftazidime: 512 μg/mL, tobramycin: 512 μg/mL) and phages (vB_PaMB13: 5.5 × 10^13^ PFU/mL, vB_PaMB17: 3.2 × 10^13^ PFU/mL) were used in the synergy tests. The combined effect of phage and antibiotic is represented in Table 1.

The minimum inhibitory concentration (MIC) value of ceftazidime for P12 bacteria decreased from 32 to 8 with each co-administration of phage-antibiotic. The FICi value was calculated as 0.25 and 0.406 for vB_PaMB13 and vB_PaMB17 phage, respectively. The MIC value of tobramycin for P12 bacteria decreased from 256 to 16 and 4 with the co-administration of the antibiotic and phage (vB_PaMB13 and vB_PaMB17 phage, respectively). The FICi value was calculated as 0.0125 and 0.0218 for each phage administration.

For the P6 isolate, a 514-fold decrease was observed in the MIC of Ceftazidime (from 8092 µg/mL to 16 µg/mL) and 3 log decrease in vB_PaMB13 phage (from 10^12^ PFU/mL to 10^9^ PFU/mL). A 64-fold decrease was observed in the MIC of Ceftazidime (from 8092 µg/mL to 128 µg/mL) and nearly one log decrease in vB_PaMB17 (from 10^12^ PFU/mL to 10^11^/64 PFU/mL). The tobramycin MIC of P6 bacteria decreased almost 20-fold with each phage application. The vB_PaMB13 phage can be used at nearly 3-log lower concentrations when combined with antibiotics.

The ceftazidime MIC value for the P24 bacteria remained unchanged when the vB_PaMB13 phage was used alongside it. The vB_PaMB13 phage was found to have an additive synergistic effect with ceftazidime. However, lower concentrations of the phage can be used in combination with the antibiotic. The MIC of the antibiotic used decreased fourfold when vB_PaMB17 phage was used in combination with ceftazidime. Similarly, the co-use model was found to allow a decrease of approximately 5 log in the phage concentration. The FIC values were calculated as 1 and 0.25 for the vB_PaMB13 and vB_PaMB17 phages, respectively.

Consequently, synergy is detected with most phages and tobramycin or ceftazidime against *P. aeruginosa.* In both antibiotic classes, the vB_PaMB13 phage is the most effective at low concentrations.

### 3.3. In Vivo Efficacy Studies of Phage Alone and Phage-Ceftazidime (CEF) Combination Treatment in G. mellonella Model

**Figure 3:**
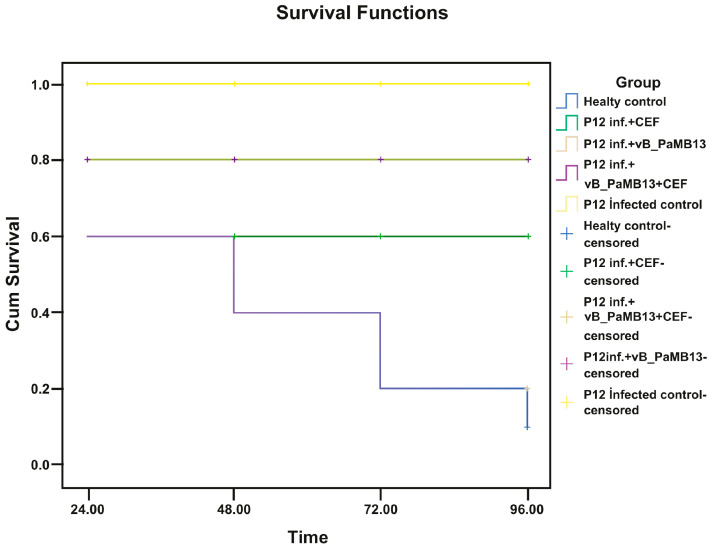
Therapeutic efficacy in *P. aeruginosa-infected Galleria mellonella* larvae under different treatments. Group 1: Healthy Control (phosphate-buffered saline). Group 2: P12 infection control (1.5 × 10^6^ CFU/mL). Group 3: vB_PaMB13 phage (10^8^ PFU/mL) alone treatment on P12 (1.5 × 10^6^ CFU/mL) infection and survival rates of *G. mellonella* larvae. Group 4: CEF (0.3 mg/mL) alone therapy on P12 (1.5 × 10^6^ CFU/mL) infection and survival rates of *G. mellonella* larvae. Group 5: Effectiveness of vB_PaMB13 phage (10^8^ PFU/mL) treatment and CEF combined therapy on P12 (1.5 × 10^6^ CFU/mL) infection and survival rates of *G. mellonella* larvae (*p* = 0.002). CEF: Ceftazidime.

**Figure 4:**
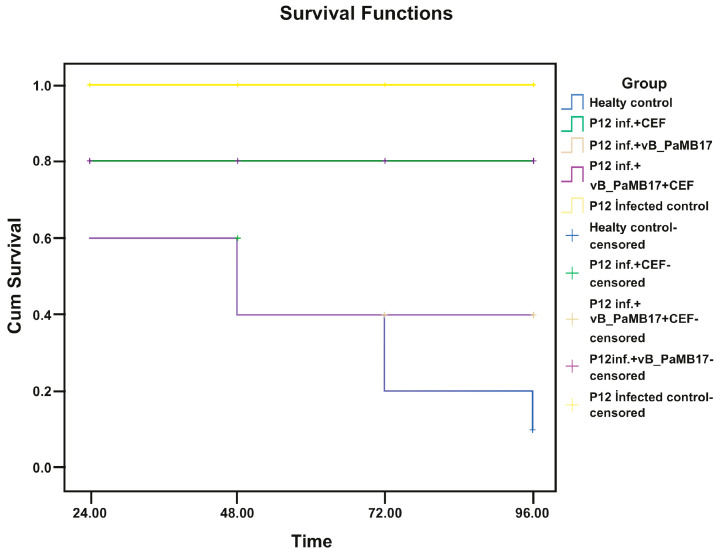
Therapeutic efficacy in *P. aeruginosa-infected Galleria mellonella* larvae under different treatments. Group 1: Healthy Control (phosphate-buffered saline). Group 2: P12 infection control (1.5 × 10^6^ CFU/mL). Group 3: vB_PaMB17 phage (10^8^ PFU/mL) alone treatment on P12 (1.5 × 10^6^ CFU/mL) infection and survival rates of *G. mellonella* larvae. Group 4: CEF (0.3 mg/mL) alone therapy on P12 (1.5 × 10^6^ CFU/mL) infection and survival rates of *G. mellonella* larvae Group 5: Effectiveness of vB_PaMB17 phage (10^8^ PFU/mL) treatment and CEF combine therapy on P12 (1.5 × 10^6^ CFU/mL) infection and survival rates of *G. mellonella* larvae (*p* = 0.008). CEF: Ceftazidime.

The in vivo activity of vB_PaMB13 and vB_PaMB17 was examined using *G. mellonella* as a model organism. PBS and vB_PaMB13, vB_PaMB17 and ceftazidime were inoculated to larvae. Firstly, in the lethal dose determination study, the determined application doses of vB_PaMB13 and vB_PaMB17 phages were tested on larvae and their 96 h vital activities were examined. As a result, the survival rate was found to be 100% for both phage groups and the treatment dose was determined accordingly. The treatment application results of the two study groups (Group A and Group B) formed afterwards are remarkable (as seen in Figure 3 and Figure 4). If Group A is examined, it is shown that the vB_PaMB13 + ceftazidime combination treatment is more successful than the treatment with vB_PaMB13 alone as a result of the single treatment with vB_PaMB13 phage and the treatment applied together with ceftazidime.

When the statistical comparison results were evaluated within themselves and evaluating the statistical data, it was observed that the combined treatment of vB_PaMB13 or vB_PaMB17 bacteriophages with ceftazidime was more effective than the single phage treatments. When the survival rates were examined as a result of the treatments, it was seen that while the survival rate in the vB_PaMB13 single phage treatment was approximately 40% (4 live 6 dead in 10 larvae) (*p* = 0.029), it was seen that the survival rate increased to 80% (8 live 2 dead in 10 larvae) in the vB_PaMB13 + ceftazidime combined treatment (*p* = 0.002).

Similarly, while the survival rate in the bacteriophage treatment with vB_PaMB17 phage was around 20% (2 live 8 dead) (*p* = 0.146), this rate increased to 60% (6 live 4 dead) in the vB_PaMB17 + ceftazidime combined treatment (*p* = 0.008).

Accordingly, the combined treatment with vB_PaMB13 + ceftazidime showed a statistically significant difference and created a good synergy (*p* = 0.003). The use of vB_PaMB13 + ceftazidime combination therapy in the treatment of P12 strain was found to be significantly effective in treating the infection.

### 3.4. Bacterial Growth Test Results in Galleria mellonella Larvae

We performed the CFU assignment to investigate the bacterial change after treatment. The colony count recorded using the CFU count method. The Dunn test was used to determine the results, and Wilcoxon signs were used for measurements for repeated measurements. Larval CFU values are given by the results of Wilcoxon’s transfer analysis. When compared across all groups, a significant difference was found (*p* = 0.002). The CFU results of the groups were analyzed, and vB_PaMB13 monotherapy CFU rates decreased from 10^8^ to 10^5^ (CFU/mL). vB_PaMB13 + ceftazidime. combination treatment CFU rates decreased from 10^8^ to 10^3^ (CFU/mL). This was statistically significant (*p* = 0.0025). vB_PaMB17 monotherapy CFU rates decreased from 10^8^ to 10^7^ (CFU/mL). vB_PaMB17 + ceftazidime combination treatment CFU rates decreased from: The number of larval CFUs decreased from 10^8^ to 10^5^ (CFU/mL)(*p* = 0.008). When these results were evaluated, the vB_PaMB13 + ceftazidime combination treatment was found to be the most effective treatment combination in killing bacteria.

## 4. Discussion

Combinations of antibiotics and lytic phages stand out as the most promising and prominent method for combating resistance. This strategy offers the potential to both reduce antibiotic use and maintain the effectiveness of existing antibiotics for longer periods by slowing the development of resistance [46].

The rising incidence of hospital and community-acquired infections caused by antibiotic-resistant *P. aeruginosa*, together with therapeutic failures associated with monotherapy, highlights the importance of identifying synergistic drug combinations.

PAS is important for developing alternative treatment approaches, particularly in the current era, because antibiotic resistance is increasing. The findings reveal that the combined use of phages and antibiotics is more effective in eliminating bacterial load than phages or antibiotics alone [47]; however, the degree of synergism may vary depending on the antibiotics and phages used [48]. It has been reported that the types and doses of phages and antibiotics are critical for achieving PAS. Different PAS application strategies are being tested, such as applying phage and antibiotic in various orders or combining them, to determine the appropriate concentration [49].

In our study, we evaluated the synergistic effects of two different phages combined with ceftazidime and tobramycin against *P. aeruginosa*. Both phages demonstrated a synergistic interaction with each antibiotic, and this effect was not only observed in vitro but also confirmed in vivo using the *G. mellonella* infection model. These findings are significant in that specific phage-antibiotic combinations may be highly effective against resistant strains.

In the study by Luo et al. (2022), in vitro time-kill assays demonstrated that the combination of phage YC#06 with antibiotics such as chloramphenicol, imipenem, and cefotaxime resulted in PAS, effectively lowering the antibiotic concentrations required to achieve bacterial inhibition compared with antibiotics alone [50].

Holger et al. (2022), in their study investigating the combined efficacy of phages with meropenem or ciprofloxacin against two MDR *P. aeruginosa* isolates, reported that phages and meropenem exhibited synergistic effects against both isolates [3]. The triple combination—phage-meropenem-colistin and phage-ciprofloxacin-colistin—resulted in the most significant CFU reduction for strains R9316 (3.50 log_10_ CFU ml^−1^) and R10266 (4.50 log_10_ CFU ml^−1^) respectively.

In another study by Chan et al. (2016), phage selection experiments conducted on MDR *P. aeruginosa* strains demonstrated that bacterial susceptibility to antibiotics could be restored [48]. Specifically, it was observed that bacteria undergoing evolutionary changes under bacteriophage pressure exhibited disruptions in some mechanisms responsible for resistance—particularly efflux pumps and outer membrane structures. As a result of these changes, bacteria have become more susceptible to various classes of antibiotics. These findings suggest that phage selection can be a powerful tool for evolutionary pressure to restore antibiotic susceptibility.

The combined use of phages and antibiotics can have different outcomes. The two agents may act synergistically, producing effects far greater than those of either agent alone; alternatively, the combined effect may remain unchanged. Finally, they may antagonize each other’s effects, resulting in no effect [51]. Therefore, in vitro evaluations of phage-antibiotic synergy are necessary before treatment to select synergistic combinations. Studies using different phages for the same antibiotic may yield contradictory results. For example, quinolones were reported to be synergistic against *P. aeruginosa* in one study, whereas an antagonistic effect was reported in another study [35,52]. Therefore, the effects of synergistic combinations should be evaluated for each phage, depending on the antibiotic’s mechanism of action, the likelihood of resistance, and the impact of the host environment on PAS activity.

There are studies investigating the mechanisms by which antibiotics affect phage lytic activity processes. It has been reported that phage combinations allow for easier cell lysis because antibiotics acting on the cell wall (such as ceftazidime) create a fragile barrier. The synergism and antagonism observed with membrane-disrupting colistin may be due to the drug’s complexity or its primary interaction with lipopolysaccharide (LPS), which causes cell membrane instability [53]. Protein synthesis inhibitors such as streptomycin likely inhibit phage production, suggesting that streptomycin inhibits topoisomerases in both bacteria and phages. Similarly, it has been reported that phages and antibiotics can help control bacterial proliferation and antibiotic resistance by targeting different bacterial receptors [54]. Furthermore, as bacteria develop resistance to one agent, their altered components or functions can make them more vulnerable to other agents [55].

Therapeutic phages belong to twelve different genera: *Pakpunavirus*, *Pbunavirus*, *Phikzvirus* and *Nankokuvirus* (myovirus morphotype); *Litunavirus*, *Bruynoghevirus*, *Paundecimvirus* and *Phikmvvirus* (podovirus morphotype); *Septimatrevirus* and *Nipunavirus* (siphovirus morphotype); and *Perrunavirus* and *Cystovirus* (enveloped, spherical or icosahedral virion morphotype) [56].

The *Pbunavirus* genus, the most comprehensively sequenced group of phages targeting *Pseudomonas*, was recently approved by ICTV taxonomy. These phages stand out due to their high lytic activity and ease of production [57]. Weiner et al. reported that nebulised phages prepared using a three-phage cocktail containing *Pbunavirus* reduced the sputum density of *P. aeruginosa* in patients with cystic fibrosis [58]. Another study reported that the LysN1 phage reduced biofilms by 80% [59]. In our study, vB_PaMB13 phage was preferred for use in in vivo studies as an effective *Pbunavirus* member with myovirus morphology. Additionally, vB_PaMB17 is not genomically characterized so far, thus the safety of the phage is not fully available.

Studies specifically on *P. aeruginosa* have reported that the effectiveness of phage-antibiotic combinations may be due to functional changes explained by phage weakening of the cell wall structure. For example, in a study by Uchiyama et al. (2018), KPP22, a phage from the *Pbunavirus* family, synergized with piperacillin and ceftazidime, causing a weakening of the *P. aeruginosa* cell wall [60]. This situation supports the notion that phages increase antibiotic susceptibility by disrupting the bacterial cell wall structure.

In the study conducted by Engeman et al. (2021), treatment with ceftazidime (CAZ), meropenem, gentamicin, or ciprofloxacin in the presence of phages increased the number of *P. aeruginosa* strains susceptible to these antibiotics by 63%, 56%, 31%, and 81%, respectively [61]. They reported that using *P. aeruginosa* phages in combination with different classes of antibiotics was not only effective but also synergistic in reducing bacterial populations, resulting in the resensitization of MDR *P. aeruginosa* to antibiotics. The study also showed that bacteria remaining in mouse wounds after combination therapy had mutations in genes associated with drug efflux pumps, which are linked to virulence. In contrast, they found that bacteria remaining in wounds treated with phages alone had mutations in phage receptors, making them resistant to phage infection. Notably, none of these mutations associated with phage resistance were detected in bacteria from the combination therapy group.

Taken together, our study reinforces the potential of phage-antibiotic synergy as an effective strategy to improve treatment outcomes and reduce bacterial resistance. However, our study has several limitations that require further investigation. These include the use of a single bacterial strain (P12), the absence of a study on resistance development, and the lack of pharmacokinetic/pharmacodynamic data. Further investigations focusing on optimizing dosing regimens, elucidating the mechanistic underpinnings of synergy, and testing in higher-order animal models will be essential to advance these findings toward clinical application.

## Figures and Tables

**Table 1:** The MICs of antibiotics (ceftazidime and tobramycin) and the effective concentration of phages vB_PaMB13 and vB_PaMB17 (PFU/mL) against bacteria (P12, P6, and P24).

Bacteria	Antibiotic	Alone	Combination	FICi Value	Comments
P12	Ceftazidime	32	8	0.25	Synergy
	vB_PaMB13	10^12^	10^8^/16		
	Ceftazidime	32	8	0.406	Synergy
	vB_PaMB17	10^7^	10^8^/64		
	Tobramycin	256	16	0.0125	Synergy
	vB_PaMB13	10^12^	10^9^/64		
	Tobramycin	256	4	0.0218	Synergy
	vB_PaMB17	10^8^	10^7^/16		
P6	Ceftazidime	8092	16	0.0157	Synergy
	vB_PaMB13	10^12^	10^9^		
	Ceftazidime	8092	128	0.126	Synergy
	vB_PaMB17	10^12^	10^11^/64		
	Tobramycin	>640	16	0.0157	Synergy
	vB_PaMB13	10^12^	10^9^/8		
	Tobramycin	>640	32	0.34	Synergy
	vB_PaMB17	10^9^	10^10^/32		
P24	Ceftazidime	8	8	1	Additive effect
	vB_PaMB13	10^12^	10^8^/512		
	Ceftazidime	8	2	0.25	Synergy
	vB_PaMB17	10^11^	10^6^/8		
	Tobramycin	2	0.5	0.25	Synergy
	vB_PaMB13	10^12^	10^4^/512		
	Tobramycin	2	0.5	0.25	Synergy
	vB_PaMB17	10^11^	10^4^/512		

## Data Availability

The sequence generated in the present work has been submitted to Genbank with PX353015 accession number.

## Supplementary Materials

The following supporting information can be downloaded at: https://www.mdpi.com/article/10.3390/pathogens14121248/s1, Table S1. Host range analysis of isolated phages. Figure S1. VIRIDIC heatmap depicting intergenomic similarities between vB_PaMB13 and selected genomes. Similarity percentages were shown both numerically and via color coding. Table S2. Table indicating the genus and species clusters analyzed by VIRIDIC.
