# Supplementary material for: Characterisation of a Novel Pseudomonas Phage and Its Effect on the Survival of Galleria mellonella Larvae
Source: Pathogens. 2025 Dec 6;14(12):1248. doi: 10.3390/pathogens14121248 (PMC12735786; doi:10.3390/pathogens14121248)
Supplement: Supplementary file 1 [file pathogens-14-01248-s001.zip › pathogens-3959969-supplementary.pdf]

Characterisation of a Novel *Pseudomonas* Phage and Its Effect on the Survival of *Galleria mellonella* Larvae

Sukran Ozturk [1,*], Hilal Basak Erol [2,*], Banu Kaskatepe [2] and Wan-Ting Huang [3]

[1] Department of Pharmaceutical Microbiology, Faculty of Pharmacy, Zonguldak Bulent Ecevit University, 67100 Zonguldak, Turkey
[2] Department of Pharmaceutical Microbiology, Faculty of Pharmacy, Ankara University, 06100 Ankara, Turkey; bkaskatepe@ankara.edu.tr
[3] Precise Health SA, C/O The Ark Foundation, Rue de L'Industrie 23, 1905 Sion, Switzerland; wan-ting@precisehealth.io
* Correspondence: sukran.ozturk@beun.edu.tr (S.O.); hcuhadaroglu@ankara.edu.tr (H.B.E.)

Table S1. Host range analysis of isolated phages.

Phage / Bacteria	vB_Pa MB1	vB_Pa MB2	vB_Pa MB3	vB_Pa MB4	vB_Pa MB5	vB_Pa MB6	vB_Pa MB7	vB_Pa MB8	vB_Pa MB9	vB_Pa MB10	vB_Pa MB11	vB_Pa MB12	vB_Pa MB13	vB_Pa MB14	vB_Pa MB15	vB_Pa MB16	vB_Pa MB17	vB_Pa MB18
**P1**	+	+	+++	+++	+++	+++	-	-	++	-	++	+++	+	-	-	+++	-	-
**P2**	-	-	+	++	+++	+++	-	-	+	-	-	-	+	+	-	+	+	-
**P3**	-	++	-	+	++	+	+++	+++	+	-	-	+	+++	+++	-	+	+++	++
**P4**	-	-	+++	+++	+++	+++	-	-	++	-	+	++	+	-	-	+++	+	-
**P5**	-	-	-	-	+	-	++	-	-	+	++	-	+	-	+	+	++	+
**P6**	+	+	-	-	-	-	+++	+++	-	-	+	-	+	+	+	+	+++	+
**P7**	-	-	-	-	-	-	+++	++	-	-	-	+	+	+	+	-	++	+
**P8**	-	+	-	+	+	+	+++	++	-	-	+	+	+	+	+	+	++	+
**P9**	-	+	-	+	-	+	++	++	-	+	+	+	+	+	+	-	+	-
**P10**	+	+	+	-	-	-	+	+	-	-	-	-	+	-	-	-	+	-
**P11**	-	+	++	+++	+++	+++	+	-	-	+	+	++	+	-	-	+++	-	-
**P12**	+	+	+	+	-	+	++	++	-	+	+	+	+	+	+	-	++	+
**P13**	-	++	+	-	++	-	+++	+++	-	-	+	-	++	++	-	-	++	-

**P14**	+	++	-	-	+++	-	+++	+++	+++	-	-	+++	+++	++	-	+	+++	++
**P15**	-	+++	-	+	+	-	+++	+++	-	-	-	+	+++	++	-	+	++	+
**P16**	-	+	+	-	-	-	+++	+++	-	-	+	++	+++	-	+++	+	+++	++
**P17**	+	+	+	-	-	-	++	++	-	-	+	-	+	+	+	-	+	+
**P18**	-	+	+++	+++	+++	+++	-	-	+	-	++	++	+	-	-	+++	-	-
**P19**	-	-	+	+++	+++	+++	-	-	++	-	+	++	+	-	-	+++	-	-
**P20**	+	-	-	-	-	+	-	-	-	-	-	-	+	-	-	-	+	-
**P21**	+	+	+	+	+	+	+	+	-	-	-	-	-	-	-	-	-	-
**P22**	-	+++	-	++	++	+	+++	+++	-	-	-	-	+++	+++	-	+	+++	++
**P23**	-	+++	-	++	++	+	+++	+++	-	-	-	-	+++	+++	-	+	+++	++
**P24**	-	++	-	+	-	-	++	++	-	-	+	-	+	-	-	-	+	+
**P25**	-	-	-	+	+	-	++	++	-	-	-	+	+	+	-	-	++	+
**P26**	-	-	+++	+++	+++	++	-	-	+	-	+	+++	++	+	-	+++	+	-
**P27**	-	+++	+	+	++	+	+	-	-	-	-	-	++	++	-	-	++	-
**P28**	-	-	+	-	-	+	-	-	-	+	+	++	++	-	-	-	+	++
**P29**	-	++	-	+	++	+	+++	+++	-	-	-	+	+++	+++	-	+	+++	++
**Host bacteria**	+	+++	+	+	++	++	++	++	+	-	+	+	++	+	++	++	++	+
**Lytic Effect%**	30	70	53.3	66.6	66.6	63.3	73.3	63.3	30	16.6	56.6	60	93.3	60	30	60	83.3	56.6

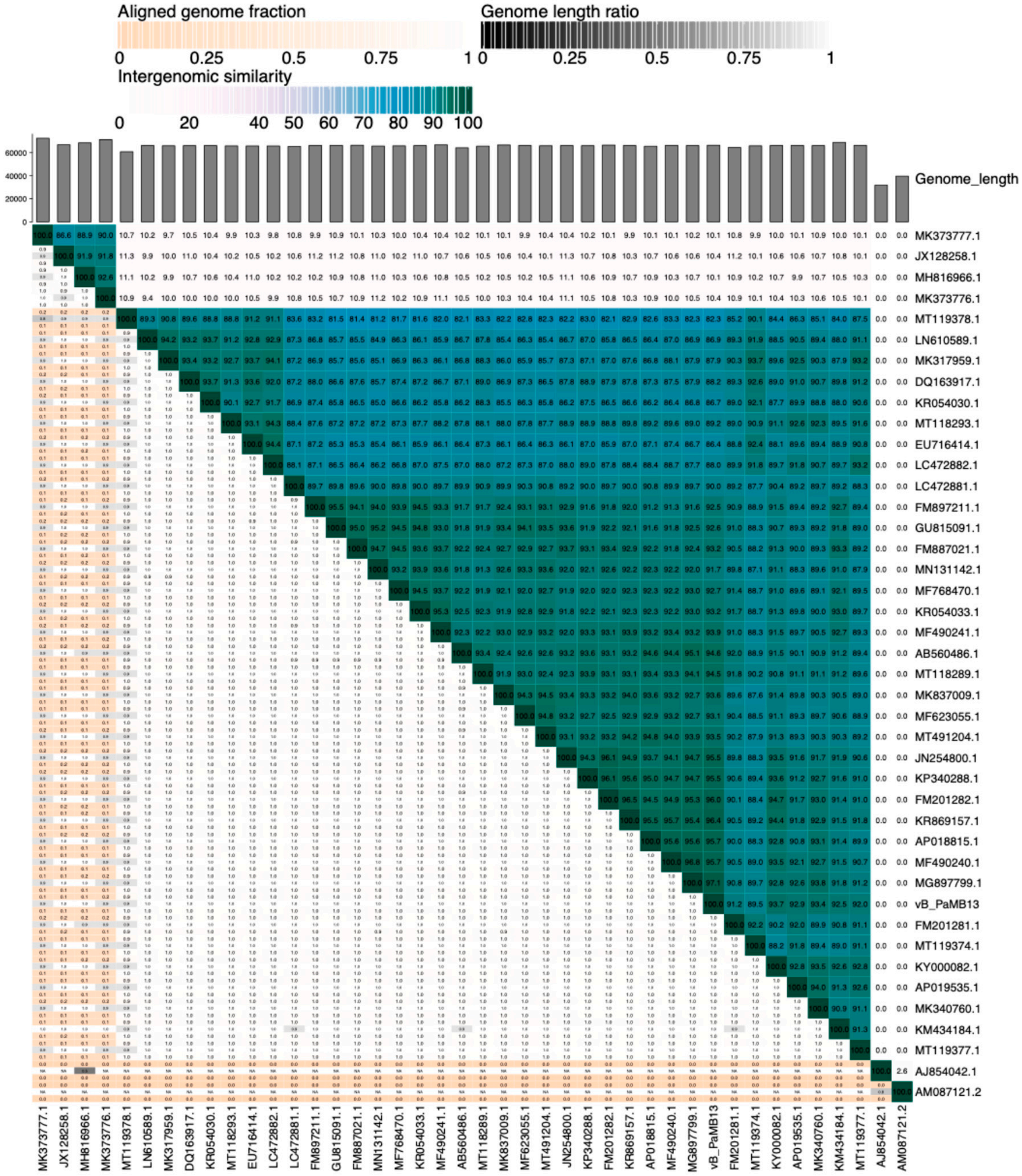

Figure S1. VIRIDIC heatmap depicting intergenomic similarities between vB_PaMB13 and selected genomes. Similarity percentages were shown both numerically and via color coding.

Table S2. Table indicating the genus and species clusters analyzed by VIRIDIC.

Genome	species cluster	genus cluster	species
MK373777.1	27	4	Wifcevirus WFC
JX128258.1	13	4	Wifcevirus ECML117
MH816966.1	23	4	Wifcevirus FEC19
MK373776.1	26	4	Wifcevirus WFH
MT119378.1	34	1	Pbunavirus datas
LN610589.1	20	1	Pbunavirus Ab28
MK317959.1	24	1	Pbunavirus EPa61
DQ163917.1	6	1	Pbunavirus F8
KR054030.1	15	1	Pbunavirus DL60
MT118293.1	31	1	Pbunavirus Epa14
EU716414.1	7	1	Pbunavirus PB1
LC472882.1	19	1	Pbunavirus R26
FM201281.1	8	1	Pbunavirus LBL3
MT119374.1	32	1	Pbunavirus antinowhere
MT119377.1	33	1	Pbunavirus crassa
AP019535.1	5	1	Pbunavirus PA01
MK340760.1	25	1	Pbunavirus S1
LC472881.1	18	1	Pbunavirus R12
KM434184.1	14	1	Pbunavirus PS44
KY000082.1	17	1	Pbunavirus PA5
JN254800.1	12	1	Pseudomonas virus NH4
KP340288.1	9	1	Pbunavirus KTN6
FM201282.1	9	1	Pbunavirus LMA2
KR869157.1	9	1	Pbunavirus DP1
AP018815.1	4	1	Pbunavirus PaGU11
MF490240.1	4	1	Pbunavirus E217
**vB_PaMB13**	**4**	**1**	
MG897799.1	4	1	Pbunavirus LS1
FM897211.1	11	1	Pbunavirus pv141
GU815091.1	11	1	Pseudomonas virus JG024
FM887021.1	10	1	Pbunavirus SN
MN131142.1	29	1	Pbunavirus PA8P1
MF768470.1	22	1	Pbunavirus SL1
KR054033.1	16	1	Pseudomonas virus DL68
MF490241.1	16	1	Pbunavirus E215
MK837009.1	28	1	Pbunavirus Pa193
MF623055.1	21	1	Pbunavirus BrSP1
MT491204.1	35	1	Pbunavirus USP1
AB560486.1	1	1	Pbunavirus KPP12
MT118289.1	30	1	Pbunavirus Epa7

AJ854042.1	2	2	Deltalipothrixvirus pozzuoliense
AM087121.2	3	3	Betalipothrixvirus pozzuoliense
